# Draft Genome Sequence of *Paenarthrobacter nicotinovorans* Hce-1

**DOI:** 10.1128/genomeA.00727-17

**Published:** 2017-07-27

**Authors:** Jing Meng, Xiaomeng Sun, Shuaishuai Li, Hao Liang

**Affiliations:** a Jinan Maternity and Child Care Hospital, Jinan, Shandong, China; b The State Key Laboratory of Microbial Technology, Shandong University, Jinan, Shandong, China; c Marine College, Shandong University, Weihai, Shandong, China

## Abstract

*Paenarthrobacter nicotinovorans* Hce-1 is a Gram-positive obligate aerobe actinomycete. We report here the complete genome sequence of this organism. The genome has a length of 4,174,362 bp and contains 4,568 protein-coding genes, 64 tRNA operons, and 22 rRNA operons. Its GC content in the gene region is 63.4%.

## GENOME ANNOUNCEMENT

*Paenarthrobacter nicotinovorans* is a Gram-positive obligate aerobe that is ubiquitous and has survivability in soils and extreme environments (1). *Paenarthrobacter nicotinovorans* has been widely used in industrial production, such as degradation of nicotine, desulfurization, removal of phosphorus and heavy metals in sewage, nitrogen fixation, and degradation of aniline and poly(vinyl alcohol) (2–6).

*Paenarthrobacter nicotinovorans* Hce-1, which was isolated from a polluted hyaluronic acid solution, is a bacterium that can grow at temperatures between 18°C and 40°C, with an optimum temperature for enzyme production of 30°C. Here, we present the complete genome sequence of *Paenarthrobacter nicotinovorans* strain Hce-1.

The Illumina HiSeq4000 platform at Shanghai Majorbio was utilized to sequence the genome of *Paenarthrobacter nicotinovorans* Hce-1. One 500-bp paired-end (PE) library was prepared for sequencing, generating 1,076.8 Mb of raw data (read length, 1,129,143,000 bp). The reads were adapter clipped and quality trimmed. Non-AGCT bases on the 5′ ends were removed before being cut and quality trimmed (sequencing quality values < Q20), and N-containing (>10%) reads were removed and small fragments (length, <50 bp) abandoned following adapter clipping and quality trimming. Next, the high-quality reads (971.9 Mb) were assessed using k-mer-counting tools, which indicated a genome size of approximately 5 Mb. A total of 5 Mb of error-corrected reads were assembled with SOAPdenovo version 2.04, and the partial gap and incorrect bases in the assembled result were supplemented and corrected by GapCloser version 1.12. Gene prediction was performed by Glimmer 3.02, and the predicted genes were annotated using the NCBI nr, GO, STRING, and KEGG databases. The draft genome contains 101 scaffolds covering 4,812,174 bp and 75 large contigs (>1,000 bp), with a total length of 4,795,994 bp. The *N*_50_ contig length is 240,695 bp, and the *N*_90_ contig length is 45,000 bp. In general, the genome has a length of 4,174,362 bp, contains 64 tRNA and 22 rRNA operons, and has a GC content of 63.4%.

The features of the genome indicate that *Paenarthrobacter nicotinovorans* strain Hce-1 has evolved self-protective mechanisms of microbial metabolism in diverse environments, including the metabolism of butanoate, 2-oxocarboxylic acid, methane, and propanoate and the degradation of benzoate.

The results of gene prediction showed that 323 genes are involved in the biosynthesis of secondary metabolites, and the potential of *Paenarthrobacter nicotinovorans* strain Hce-1 to produce secondary metabolites was analyzed with the secondary metabolites search tool antiSMASH (7).

*Paenarthrobacter nicotinovorans* strain Hce-1 also utilizes a variety of carbon sources from plants, such as fructose, galactose, mannose, sucrose, and lactose, to supply its growth.

The most important finding is that *Paenarthrobacter nicotinovorans* strain Hce-1 has evolved metabolic mechanisms to transform nicotinate and nicotinamide into NAD^+^ and NADP^+^ for participation in a series of metabolic activities in the cell. *Paenarthrobacter nicotinovorans* strain Hce-1 might be useful in processing tobacco by reducing the nicotine content. This method will not cause the loss of the smell of smoke and might also increase the utilization of tobacco resources.

### Accession number(s).

This genome shotgun project has been deposited in the DNA Data Bank of Japan (DDBJ) under the GenBank accession numbers BDDW01000001 through BDDW01000101 (101 entries).
